# Bilateral Medial Thalamotomy and Anterior Cingulotomy With Gamma Knife Radiosurgery for Refractory Pain: A Case Report

**DOI:** 10.7759/cureus.89879

**Published:** 2025-08-12

**Authors:** Jorge Torres Monterrosa, Larissa Merlos Salazar, Carlos Tobar, Eduardo E Lovo

**Affiliations:** 1 General Practice, Universidad de El Salvador, San Salvador, SLV; 2 General Practice, Universidad Dr. José Matías Delgado, San Salvador, SLV; 3 Radiation Oncology, International Cancer Center, Diagnostic Hospital, San Salvador, SLV; 4 Neurosurgery-Gamma Knife Program, International Cancer Center, Diagnostic Hospital, San Salvador, SLV

**Keywords:** anxiety-depressive disorder, gamma cingulotomy, gamma thalamotomy, intractable pain, neuropathic pain, oncological pain, radiomodulation, radiosurgery

## Abstract

Medial thalamotomy as well as cingulotomy has been a classical neurosurgical practice for pain, and the latter has also been used for treatment-resistant psychiatric conditions, particularly affective disorders. Here, we present the case of a patient with a seven-year history of progressive refractory neuropathic pain secondary to an intramedullary tumor treated solely with radiotherapy. She had regular emergency room visits for breakthrough pain unresponsive to high-dose opioids and was eventually referred to palliative care. Due to the severe impact of chronic pain on her quality of life, she developed a comorbid anxiety-depressive disorder and was treated in the national psychiatric center for several years without improvement. With no further treatment options available, she was referred for Gamma Knife radiosurgery. She was treated with bilateral irradiation of the cingulum and medial thalamus. Prior to treatment, her Visual Analogue Scale (VAS) pain score was 9/10. One year post-treatment, the patient reported a substantial reduction in pain intensity (VAS 2/10), along with a marked improvement in quality of life, enabling her to return to work. Follow-up neuroimaging at eight and twelve months revealed a unilateral hyper-response to the treatment exclusively on the left hemisphere. This case emphasizes a possible treatment option for patients with chronic neuropathic, intractable pain for achieving effective, prolonged pain control, improved functionality, with no adverse physical effects. To the best of our knowledge, to date, a radiosurgical treatment combining cingulotomy and thalamotomy has not been previously reported.

## Introduction

Chronic pain in both oncological and non-oncological patients represents a significant burden to patients, family, and healthcare expenditures. This is because of its high impact on quality of life and its strong association with psychiatric comorbidities such as depression and anxiety. In oncological patients, it is estimated that more than two-thirds of those in advanced, metastatic, or terminal stages experience moderate to severe pain [1,2], and of all oncological patients with pain, up to 30% can be refractory to opioids [3].

The identification and selective inhibition of specific neural circuits involved in pain as well as psychiatric disorders may offer different therapeutic targets for symptom relief and improved clinical outcomes [4]. One such target is the cingulum bundle (CB), a white matter tract known for its integral role in affective processing and pain response. The CB is involved in mediating functions like emotional reaction to pain and suffering in the brain's pain matrix [5,6]. This pain matrix encompasses the complex multidimensional experience of pain, which involves three components: the somatosensory perception of noxious stimuli; the activation of emotional and motivational systems that assign negative affect and unpleasant perception; and the cognitive appraisal and modulation of the pain experience [7].

As a result, the CB has been increasingly investigated as a target for functional neurosurgical interventions, including a variety of lesioning techniques and radiosurgical approaches, with variable success rates and complications depending on the pathology and technique used [4,8-13]. Despite promising outcomes and its non-invasive, outpatient-based nature, cingulotomy with radiosurgery remains scarce in the literature, with only a limited number of case series focusing on psychiatric indications [4,11]. On the other hand, irradiation to the medial thalamus has been well described in the management of chronic pain in both oncologic and non-oncologic pain by our group and others [14-18], and yet despite its potential efficacy, it is also underutilized, with a very small number of patients treated worldwide.

In this report, we present a case of combined irradiation of the cingulum and bilateral thalamotomy with Gamma Knife (GK) for the treatment of comorbid anxiety-depressive disorder and refractory, neuropathic, oncological, and treatment-derived pain.

## Case presentation

The retrospective single case review and report were approved by the ethics committee of our institution, the International Cancer Center, San Salvador, under the number CECIC2025/001. Before treatment, the case was discussed with the referring institution, algology, neurosurgery, radiation oncology, and neuropsychiatry of our institution. The patient, along with her caretaker, was provided with a proper informed consent that was signed before treatment. The 45-year-old woman presented in June 2024 with a seven-year history of secondary progressive, refractory neuropathic pain localized to the right upper limb (RUL), left lower limb (LLL), and skin region of the midline cervical and thoracic spine from C6 to T8. She described stabbing back pain radiating to the LLL, accompanied by paresthesia and burning-like pain in the RUL. Before the pain became refractory, she was diagnosed with an intramedullary spinal cord tumor extending from C5 to T7, presumed to be an intramedullary glioma based solely on radiological findings; no biopsy was performed. Surgical intervention was deemed nonviable by an outside institution, and she was treated with radiotherapy, receiving only 16 out of the 25 scheduled sessions, with a total radiation dose of 25.6 Gy out of the planned 40 Gy. Treatment was prematurely discontinued due to deterioration in general health quality.

One month before radiosurgical intervention, the patient was admitted to the emergency department on three separate occasions for pain crises. Her oral opioid regimen escalated to 270 morphine milligram equivalents (MME) per day, excluding rescue morphine doses and intravenous morphine infusions. The department of clinical oncology determined that no oncological systemic treatment option was viable for her case, neurosurgery considered that she would not benefit from surgical interventions, thus she was then referred to palliative care for pain management.

Due to severe neuropathic pain over the years, the patient became non-ambulatory for eight months before radiosurgical treatment, requiring a caregiver for all activities of daily living (ADLs). This loss of independence, along with persistent and poorly managed pain and crises, led to the development of a severe anxiety-depressive disorder for which she received treatment at the national psychiatric center for several years. Her baseline assessment, measured with the EuroQol-5D-5L (EQ-5D) instrument [19], was used, and permission was obtained from the EuroQol Research Foundation for the use of the instrument in this study under the registration number 75782. The EQ-5D results, prior to Stereotactic Radiosurgery (SRS), were level five for "mobility," level five for "self-care," level four for "usual activities," level five for "pain/discomfort," and level five for "anxiety/depression," proving an extremely poor quality of life and significant anxiety-depressive symptoms.

On neurological examination, the patient exhibited allodynia in the RUL, with burning pain on light palpation of the skin. Movement-related pain in the back, RUL, and LLL made her unable to stand or ambulate. Her baseline pain score on VAS was 9/10 in the RUL, LLL, and back.

No specific scale for neuropathic pain was utilized, and baseline Beck Depression Inventory (BDI) scores were not recorded.

Treatment plan

**Figure 1:**
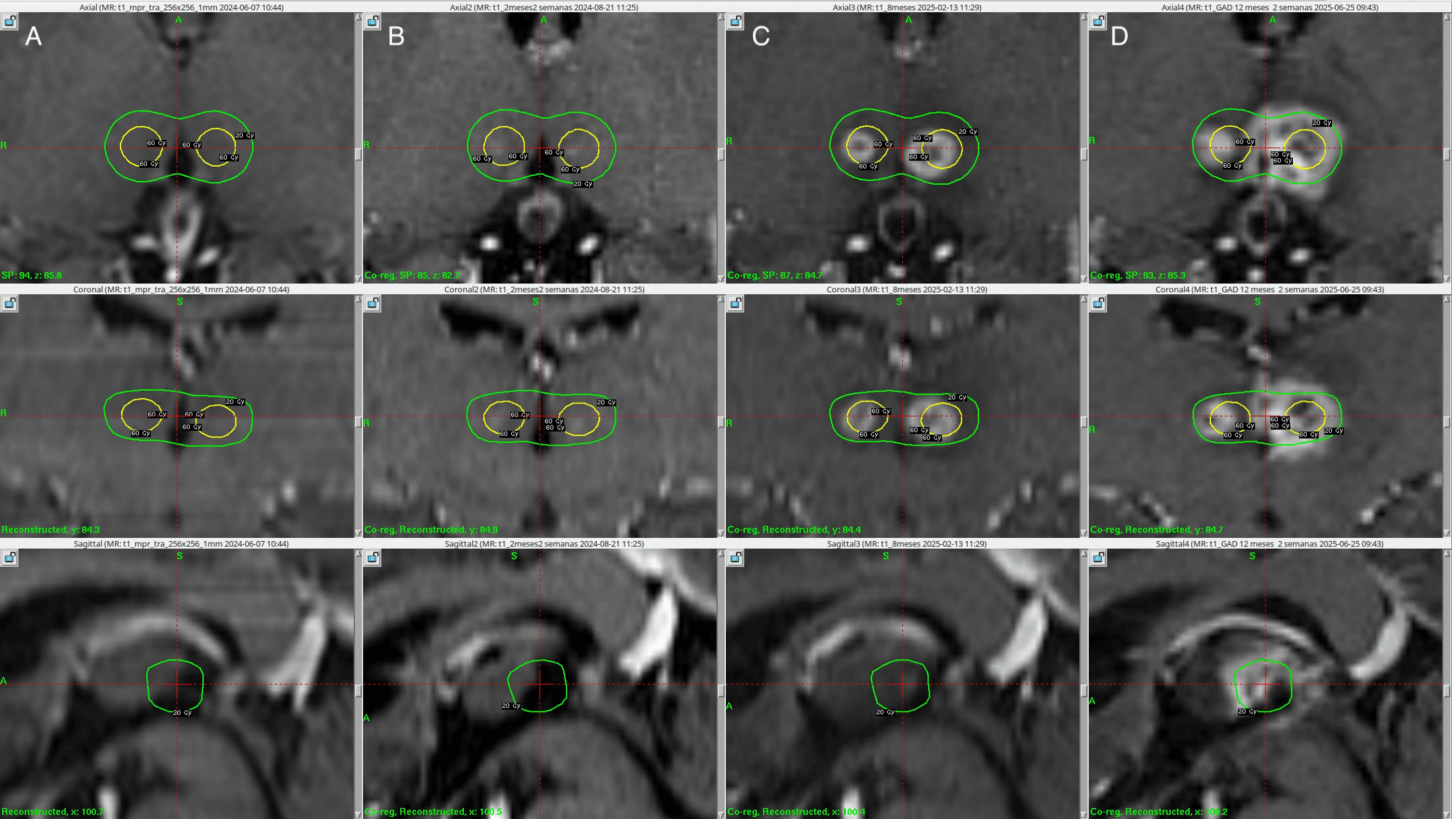
Thalamus targets at the day of treatment, two, eight, and 12 months in radiological follow up with isodose lines Column A. Three-dimensional view, axial, coronal, and sagittal of the day of treatment, prescribing 120 Gy Dmax, the internal yellow isodose line corresponds to the 60 Gy, and the outermost green isodose line to the 20 Gy. Column B. Three-dimensional view, axial, coronal, and sagittal of the two shoots at the thalamus, at two months post-treatment. A slight contrast-enhancing lesion was evidenced bilaterally inside the yellow isodose line that corresponds to the 60 Gy. Column C. Three-dimensional view, axial, coronal, and sagittal of the two shoots at the thalamus, at eight months post-treatment. A leftmost lesion was evidenced outside the internal yellow isodose line that corresponds to the 60 Gy, but within the green isodose line to the 20 Gy. Column D. Three-dimensional view, axial, coronal, and sagittal of the two shoots at the thalamus, at 12 months post-treatment. An increase in lesion size was evidenced on the left thalamus, extended outside the green isodose line to 20 Gy.

**Figure 2:**
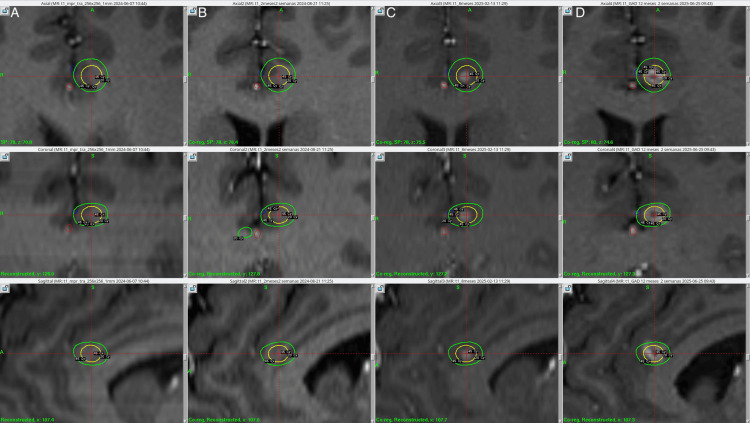
Left anterior cingulum bundle targets at the day of treatment, two, eight, and 12 months in radiological follow up with isodose lines Column A. Three-dimensional view, axial, coronal, and sagittal of the left shoot at the anterior cingulum bundle, the day of treatment, prescribing 90 Gy Dmax, the internal yellow isodose line corresponds to the 45 Gy, and the outermost green isodose line to the 20 Gy. The anterior cerebral artery branches were outlined in blue (left) and red (right). Column B. Three-dimensional view, axial, coronal, and sagittal of the left shoot at the anterior cingulum bundle, no radiological findings were observed at two months post-treatment. Column C. Three-dimensional view, axial, coronal, and sagittal of the left shoot at the anterior cingulum bundle at eight-month follow-up. A contrast-enhancing lesion was evidenced within the yellow isodose line of 45 Gy. Column D. Three-dimensional view, axial, coronal, and sagittal of the left shoot at the anterior cingulum bundle at 12 months follow-up. A larger lesion was observed extending outside the yellow isodose line corresponding to the 45 Gy, but within the green isodose line of 20 Gy.

**Figure 3:**
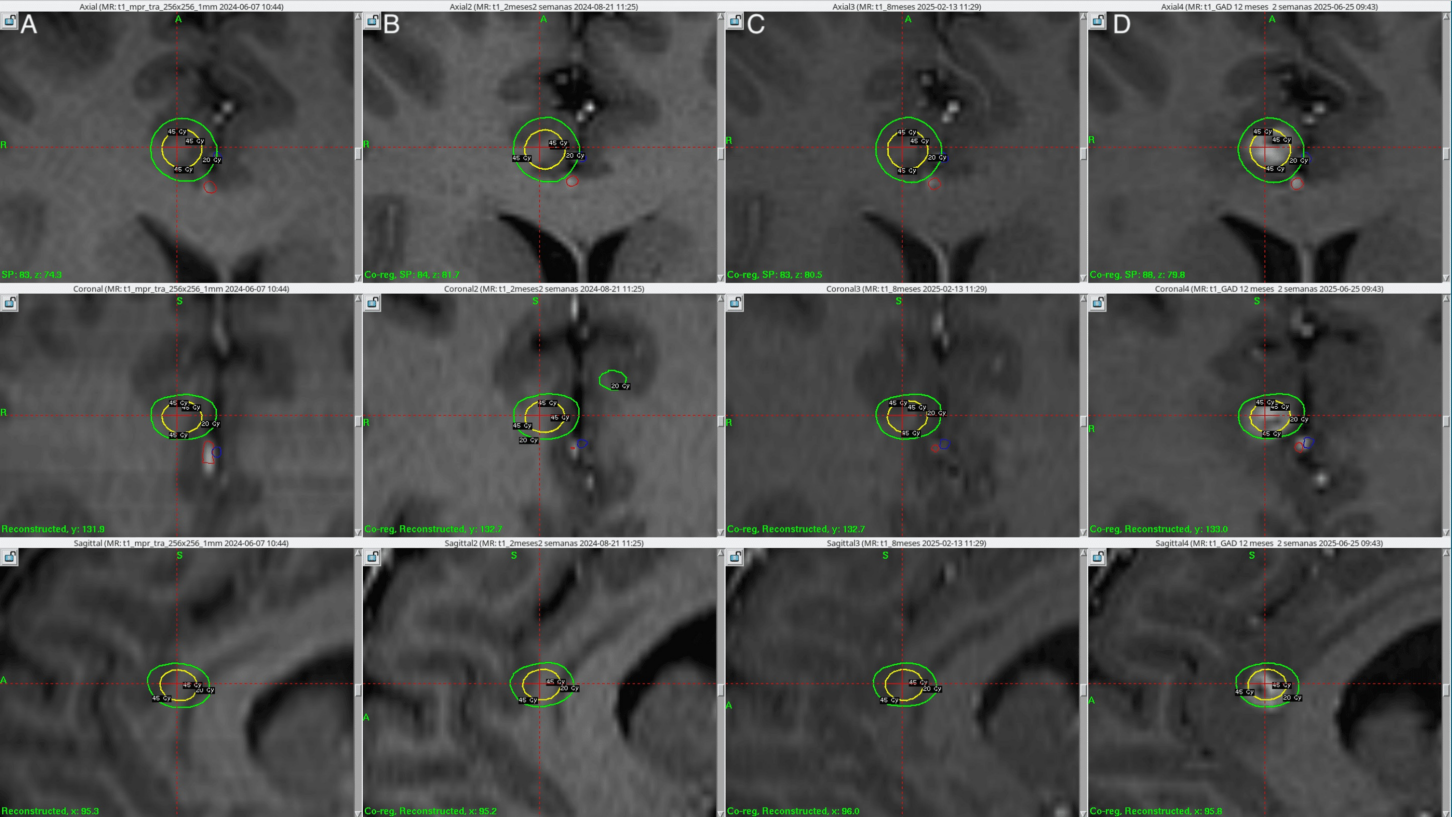
Right anterior cingulum bundle targets at the day of treatment, two, eight, and 12 months in radiological follow up with isodose lines Column A. Three-dimensional view, axial, coronal, and sagittal of the right shoot at the anterior cingulum bundle, the day of treatment, prescribing 90 Gy Dmax, the internal yellow isodose line corresponds to the 45 Gy, and the outermost green isodose line to the 20 Gy. The anterior cerebral artery branches were outlined in blue (left) and red (right). Column B. Three-dimensional view, axial, coronal, and sagittal of the right shoot at the anterior cingulum bundle, no radiological findings were observed at two months post-treatment. Column C. Three-dimensional view, axial, coronal, and sagittal of the right shoot at the anterior cingulum bundle, no radiological findings were observed at eight months post-treatment. Column D. Three-dimensional view, axial, coronal, and sagittal of the right shoot at the anterior cingulum bundle at 12 months follow-up. A new contrast-enhancing lesion was observed extending outside the yellow isodose line corresponding to the 45 Gy but within the green isodose line of 20 Gy.

Treatment was delivered using the Leksell Gamma Knife Icon (Elekta AB, Stockholm, Sweden). The patient received a prescription dose of 45.0 Gy to the 50% isodose line, using a 4.0 mm collimator, with a single shot administered to the anterior CB bilaterally, resulting in a maximum dose (Dmax) of 90.0 Gy, the anterior cerebral arteries were defined as an organ at risk and radiation dose to a focal point was kept under 25 Gy. Additionally, a dose of 60.0 Gy to the 50% isodose line was delivered to each medial thalamus, using a 4.0 mm collimator, with a corresponding Dmax of 120.0 Gy. The target was 4 mm anterior to the posterior commissure, 4 mm laterally from the thalamic border, and 2 mm superior to the anterior commissure and posterior commissure line (ACPC). Treatment planning was performed using a stereotactic, contrast-enhanced T1-weighted MRI sequence with 1-mm slice thickness with 0 space in between. The total beam-on time was 155.9 minutes, delivered at a rate of 3.2 Gy/min. The treatment plan was approved by neurosurgery, radiation oncology, and physics. The patient tolerated the procedure well and was discharged on the same day without any complications. Figures 1-3 show the SRS treatment plan.

Treatment response

The patient underwent clinical follow-ups over a 12-month period to monitor pain intensity, opioid usage, and quality of life, using the Visual Analogue Scale (VAS) and EQ-5D instrument.

**Figure 4:**
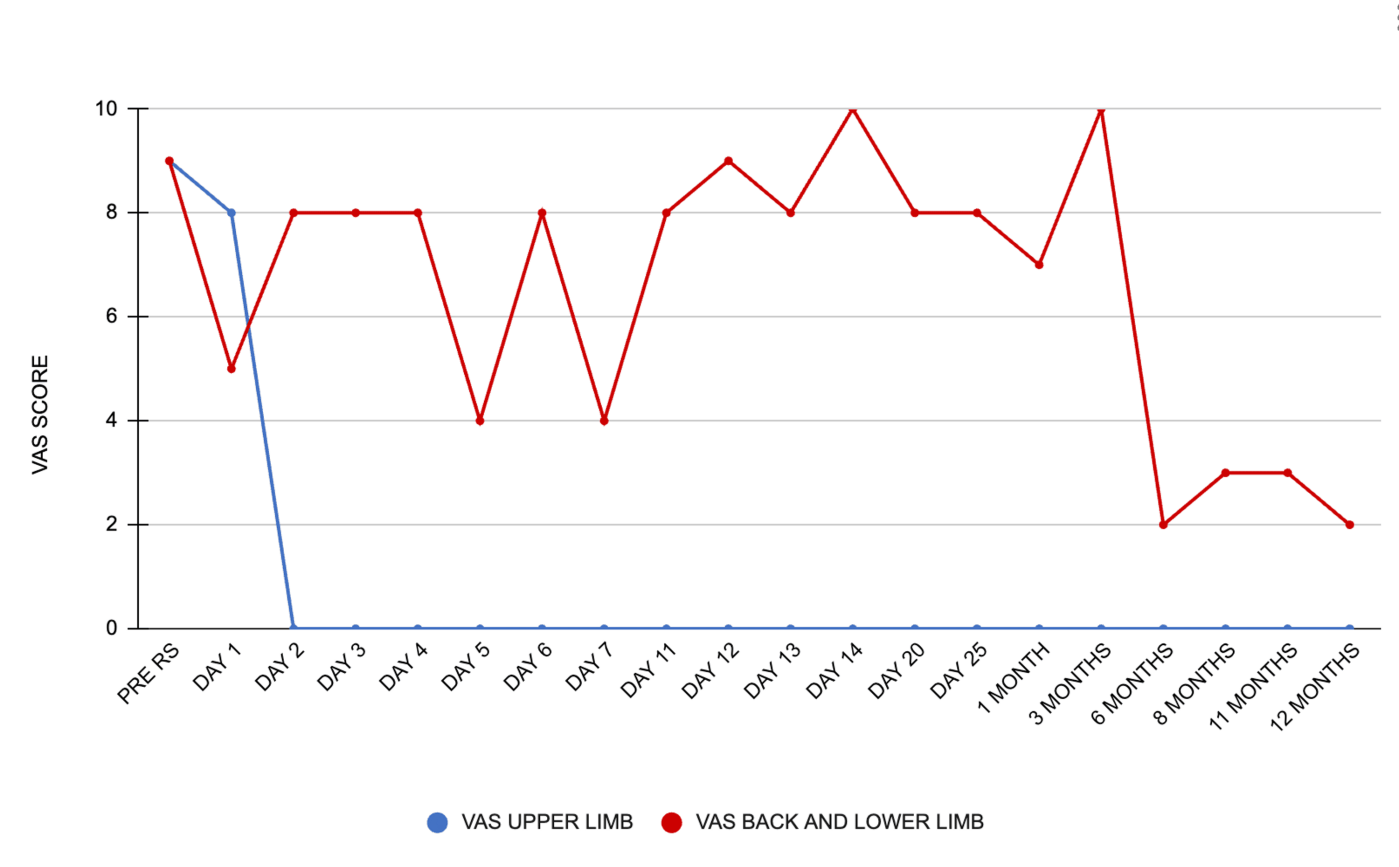
VAS score in pain assessment during follow-up Line in red line represents pain in the back and lower limb, blue line represents pain in the patient's upper limb. Plotted on the X-axis is the day before and the days or month(s) after radiosurgery; on the Y-axis is the VAS score. VAS: Visual Analogue Scale; RS: radiosurgery

Early treatment response appeared on post-treatment day one; this phenomenon has been described as radiomodulation by our group [17], which implies a substantial, more than 50% decrease in pain in a short period of time, usually less than 72 hours after treatment. She reported VAS of 5/10 in the left lower limb (LLL) and 8/10 in the right upper limb (RUL). By day two, her pain scores changed to 8/10 in the LLL and 0/10 in the RUL, the latter of which was sustained through 12 months post-treatment. However, pain intensity in the LLL fluctuated between day four and the sixth month, before stabilizing at 3/10 by the eighth month, and 2/10 in the last follow-up at 12 months, as illustrated in Figure 4.

The patient regained ambulation and was able to independently perform ADLs, resuming full-time work by the eight-month follow-up. At 11 months, she experienced a pain crisis that required rescue medication, leading to a temporary cessation of work.

**Figure 5:**
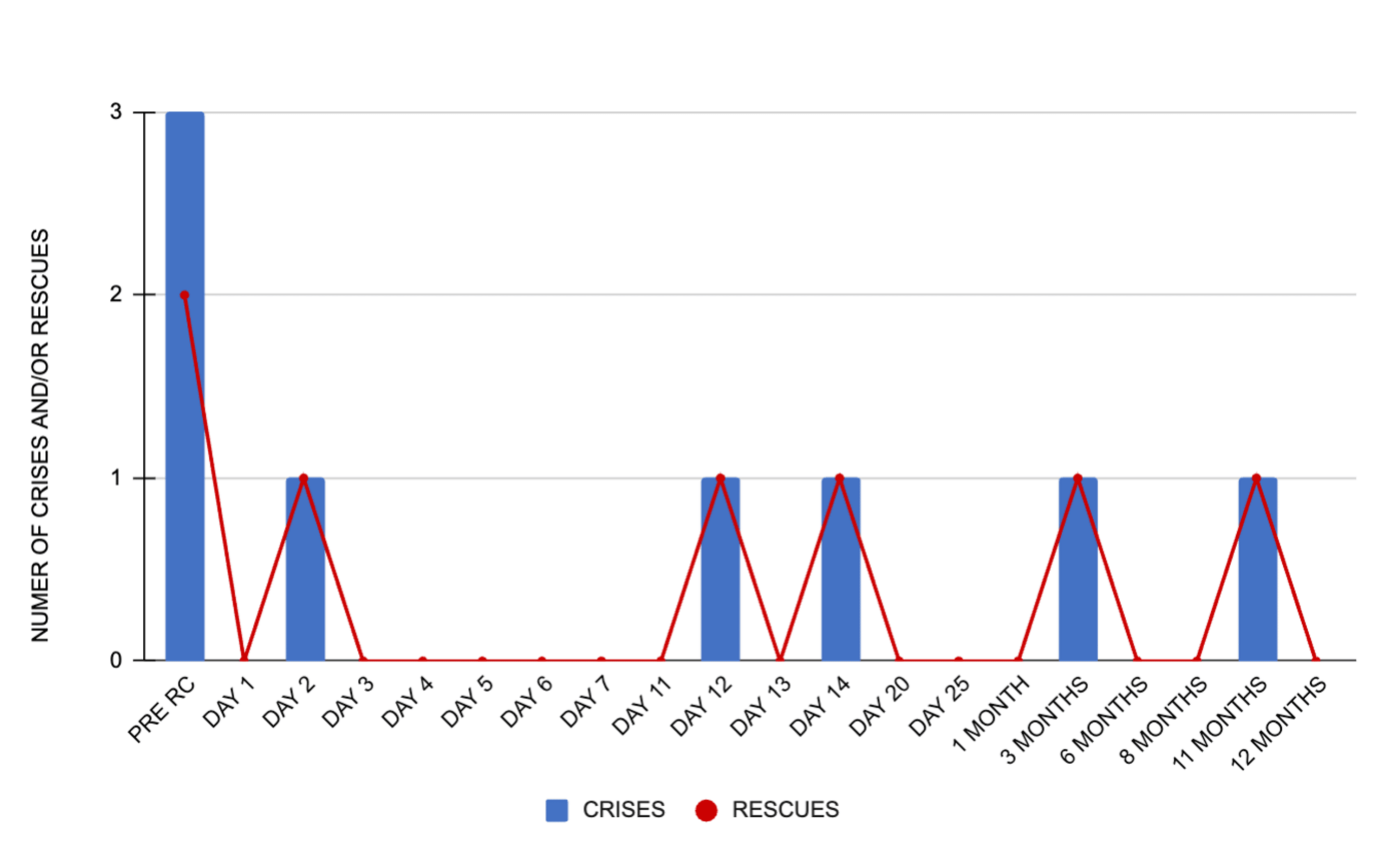
Pain crises and opioid rescue medication usage during follow-up Pain crises are represented in the blue bars, and the need for rescue medicine is represented in the red line. Plotted on the X-axis is the day before and the days or month(s) after radiosurgery; on the Y-axis is the number of crises and rescue medication usage. RS: radiosurgery

Opioid usage in morphine milligram equivalents (MME) was tracked and showed a fluctuating trend: from 270 MME pre-treatment to 216 MME at two months, 240 MME at nine months, and 453 MME at 11 months post-treatment. The dosage increase noted during the 11-month follow-up resulted from a change in the frequency of a 50 mcg fentanyl patch, from every 72 hours to every 24 hours. The surge also coincided with her father's passing. However, at the 12-month follow-up, her pain specialist returned to the original 72-hour schedule and discontinued oral tramadol because of adverse effects, leading to a 180 MME opioid usage per day at the last follow-up with good pain management. Across 12 months five pain crises were reported; nevertheless, only two required hospital admission for pain management (compared to three in the month prior to treatment), while the remaining three were managed on an outpatient basis. Rescue medication was used in the five pain crises, as shown in Figure 5.

Only two hospital admissions for pain management were required during the 12-month period, as opposed to three emergency admissions within a month before treatment.

**Figure 6:**
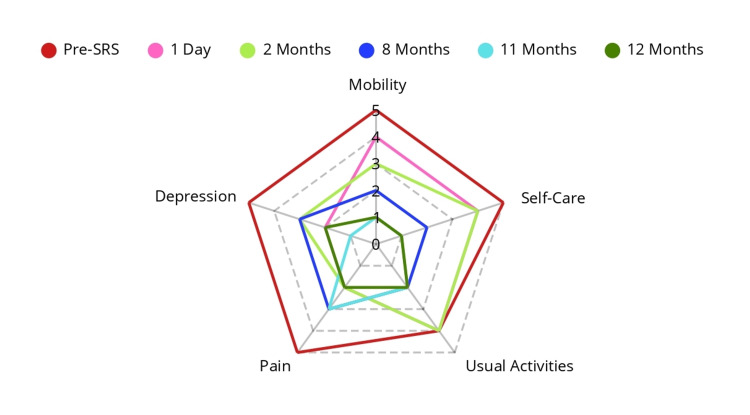
Quality of life assessments throughout treatment EQ-5D scores in quality of life assessments in follow-ups, outer red line of the pentagon on the day of treatment, resulting in a total of 24 out of 25 levels, at last follow-up in yellow at the center, giving eight out of 25 levels, representing a 66% improvement in quality of life. ED-5D: EuroQoL-5D; SRS: stereotactic radiosurgery

At the final follow-up at one year, she demonstrated substantial pain relief, improved quality of life, and reduced symptoms of anxiety and depression, as measured with the EQ-5D instrument, resulting in level one for Mobility, level one for Self-care, level two for Usual activities, level two for Pain/discomfort, and level two for Anxiety/depression, as seen in Figure 6.

A neuropsychiatric evaluation at 11 months revealed persistent depressive symptoms despite clinical improvement in pain. Using the BDI, a score of 43 was recorded, indicative of a major depressive episode, which was interfering with her daily functioning and recovery. Even though she denied apparent affective symptoms during direct questioning, the psychiatric assessment led to a modification of her pharmacological prescription, transitioning to desvenlafaxine from prior treatment with sertraline and fluoxetine.

No new neurological deficits were observed throughout the follow-up period.

**Figure 7:**
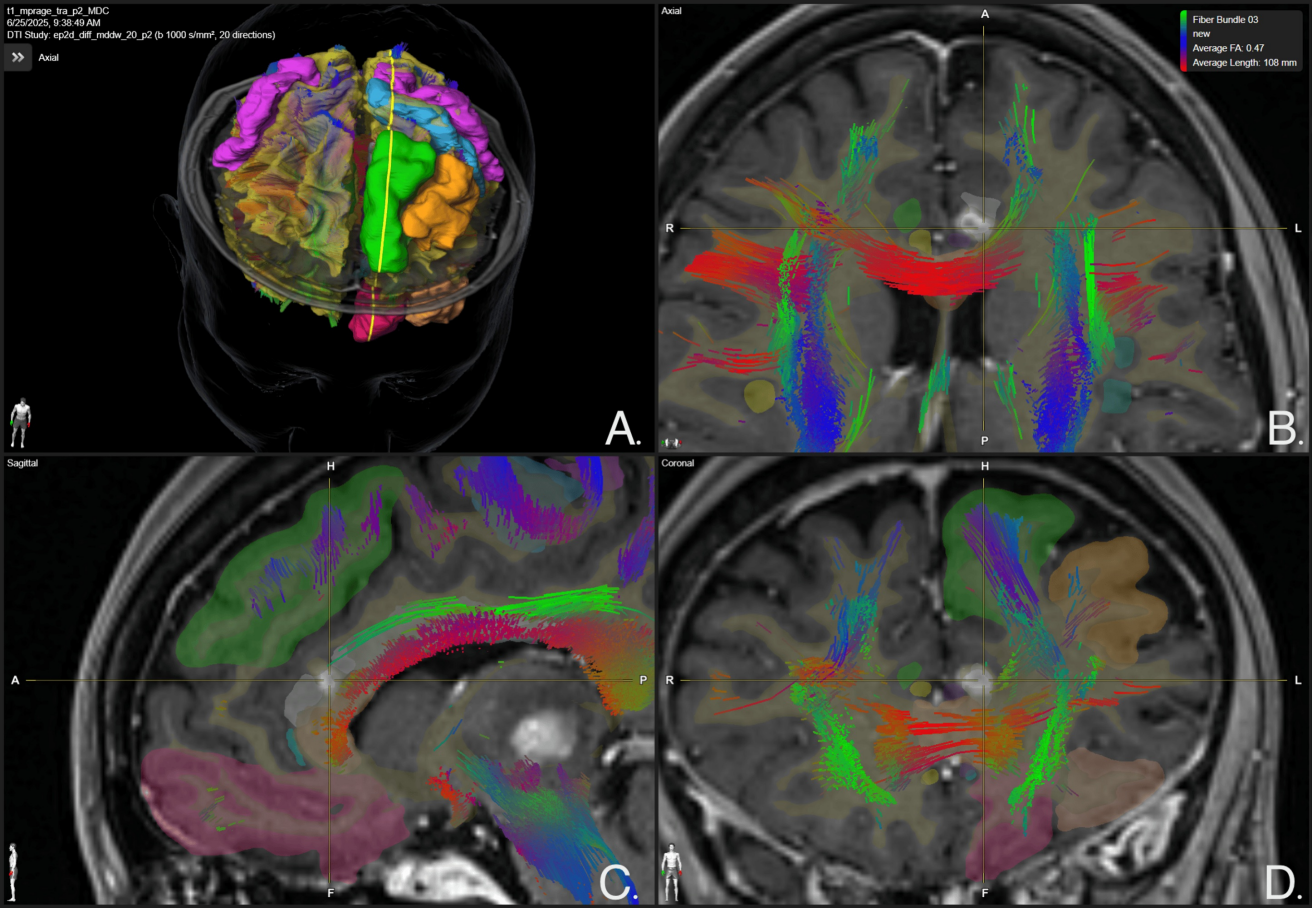
Whole brain tractography focused on the left anterior cingulum bundle lesion at 12-month follow-up Panel A. Three-dimensional reconstruction of whole brain white matter tracts. Panel B. Axial view of the brain. A disruption of white matter tracts crossing the left anterior cingulum bundle is evidenced, specifically involving the area of contrast-enhancing lesion. Panel C. Sagittal view of the brain. A disruption of white matter tracts crossing the left anterior cingulum bundle is evidenced, specifically involving the area of contrast-enhancing lesion. Panel D. Coronal view of the brain. A disruption of white matter tracts crossing the left anterior cingulum bundle is evidenced, specifically involving the area of contrast-enhancing lesion. BrainLab Elements (BrainLab, Munich, Germany) was used to generate the images.

**Figure 8:**
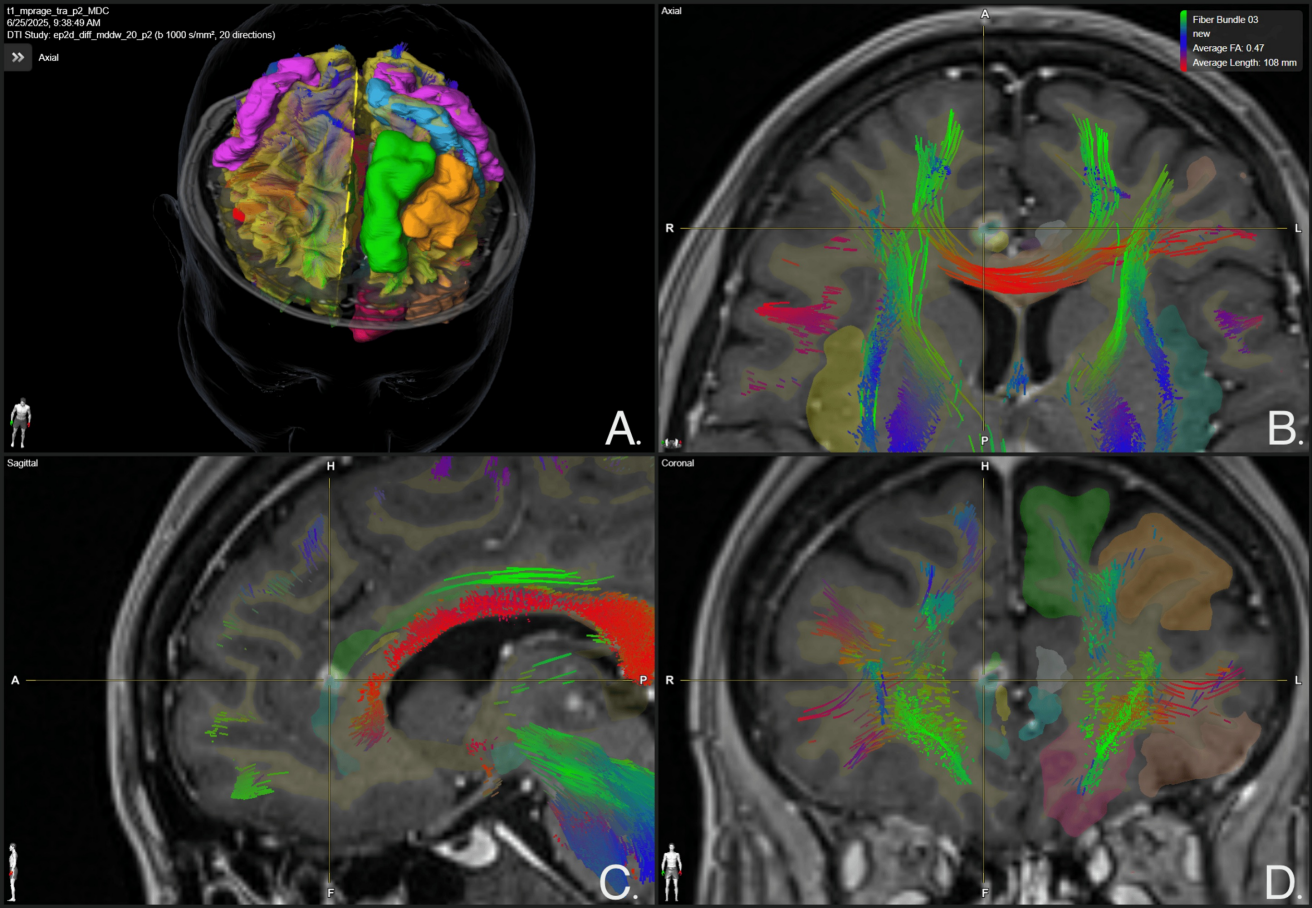
Whole brain tractography focused on the right anterior cingulum bundle lesion at 12-month follow-up Panel A. Three-dimensional reconstruction of whole-brain white matter tracts. Panel B. Axial view of the brain.  A disruption of white matter tracts crossing the right anterior cingulum bundle is evidenced, specifically involving the area of contrast-enhancing lesion. Panel C. Sagittal view of the brain.  A disruption of white matter tracts crossing the right anterior cingulum bundle is evidenced, specifically involving the area of contrast-enhancing lesion. Panel D. Coronal view of the brain.  A disruption of white matter tracts crossing the right anterior cingulum bundle is evidenced, specifically involving the area of contrast-enhancing lesion. BrainLab Elements (BrainLab, Munich, Germany) was used to generate the images.

**Figure 9:**
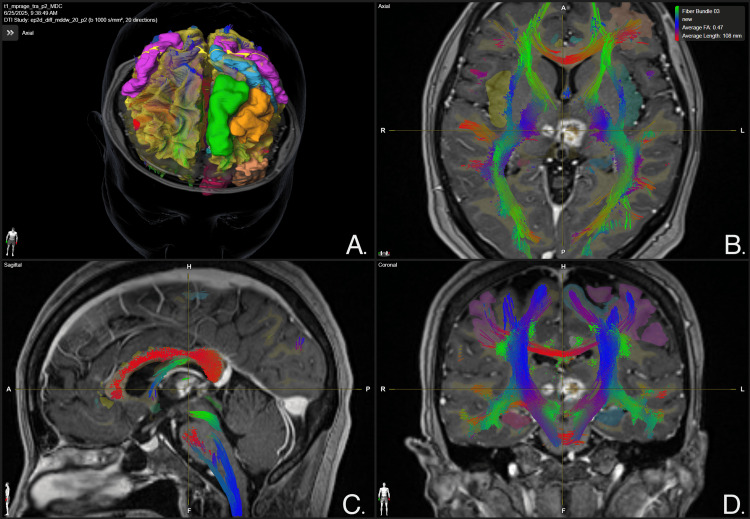
Whole brain tractography focused on thalamic lesions at 12-month follow-up Panel A. Three-dimensional reconstruction of whole brain white matter tracts. Panel B. Axial view of the brain, a leftmost disruption of white matter tracts crossing the medial thalamus is evidenced, specifically involving the area of contrast-enhancing lesions. Panel C. Sagittal view of the brain, a disruption of white matter tracts crossing the medial thalamus is evidenced, specifically involving the area of contrast-enhancing lesions. Panel D. Coronal view of the brain, a leftmost disruption of white matter tracts crossing the medial thalamus is evidenced, specifically involving the area of contrast-enhancing lesions. BrainLab Elements (BrainLab, Munich, Germany) was used to generate the images.

Post-treatment images were acquired at two, eight, and 12 months. At the two-month follow-up, MRI with contrast-enhanced T1-weighted sequencing showed slight contrast-enhancing lesions in the thalamic targeted regions bilaterally, as shown in Figure 1. Additionally, at the level of the thalamic targets, contrast enhancement was more pronounced on the left thalamus, demonstrating a hyper response to treatment of the patient's left thalamus as opposed to her right thalamus. At 12-month follow-up, a new contrast-enhancing lesion was evidenced at the right CB (Figure 3), making it consistent with a classical bilateral cingulotomy image. The left thalamic lesion continued to show a hyper-response effect, increasing in size as compared to the right thalamic lesion, which maintained its size. Despite the radiological adverse radiation effect (RARE), the patient has remained asymptomatic at last follow-up. MRI at 12 months showed contrast-enhancing lesions in the CB bilaterally, suggesting actual ablation and the classical disrupting effect of cingulotomy as well as thalamotomy, as can be seen in Figures 7-9.

Additionally, tractography studies were employed solely for post-treatment imaging evaluation and not for target selection.

## Discussion

Depression and anxiety are highly prevalent in patients with chronic pain; of these, approximately 40% of adults who suffer from chronic pain can experience clinically significant symptoms of these psychiatric disorders [20]. Such comorbidities are further associated with increased pain intensity and significant functional impairment, including disability and reduced quality of life [21]. Pain itself may act as an independent risk factor for developing mood and anxiety disorders; moderate to severe pain has been linked to elevated risks of these conditions, irrespective of prior psychiatric history [22].

The cingulate gyrus and CB are main components of the limbic system, involved in emotion regulation, cognitive processing, and the pathophysiology of mood disorders such as anxiety and depression. The anterior cingulate cortex (ACC) functions as a center for emotional processing, and hyperactivity in the ACC has been implicated in the development of anxiety and depression, especially in the context of chronic pain. As such, targeted lesioning of the ACC may improve psychiatric symptoms by disrupting maladaptive neural circuits involved in the affective component of pain and mood dysregulation [23-25].

In a tractography-based study, Sweet et al. identified region-specific connectivity patterns within the CB, implicating 11 brain areas relevant to mood disorders: the amygdala, thalamus, dorsolateral and dorsomedial frontal cortices (dlFC, dmFC), medial/central and lateral orbitofrontal cortices (m/cOFC, lOFC), ventrolateral prefrontal cortex (vlPFC), dorsal and subgenual ACC (dACC, sACC), posterior cingulate cortex (PCC), and the frontal pole (FP) [26]. Their findings confirmed that varied CB segments contribute to both dorsal and ventral emotional processing networks.

Previously, cingulotomy targets were located more posteriorly in the dorsal CB, affecting dorsal cortical circuits (dmFC, dACC, PCC). More recent approaches include targeting the subcallosal CB, which connects to ventral networks (sACC, m/cOFC). Our case employed a rostral dorsal CB target, strategically positioned to access both dorsal and ventral pathways [26].

A “more anterior” CB target has previously been recommended by Steele et al. [12], showing better outcomes for chronic pain and depression, likely due to the greater density of afferent and efferent tracts in the anterior versus posterior CB [6]. This further emphasizes the need for standardization for precise anatomical targeting when managing comorbid affective and pain conditions, and possibly the incorporation of tractographic studies and thus connectomic-based radiosurgery to further refine targets for pain at the cingulum.

Cingulotomy lesioning has a long history in treating intractable pain and psychiatric disorders. Foundational studies by Foltz and White (1962) and Ballantine et al. (1987) demonstrated that patients with high emotional reactivity and comorbid depression or anxiety responded well to cingulotomy [8,13]. The emergence of MRI-guided stereotactic lesioning further improved targeting precision and outcomes in these populations [10], establishing the CB as a neuroanatomical hub in emotional-pain processing.

Building off this evidence, our case uses GK to target the CB and medial thalamus, aiming to treat refractory neuropathic, oncologically, and treatment (radiation) derived pain that is co-occurring with anxiety-depressive disorder in a single session, an approach not previously described in the literature.

In 2008, Kim and Lee reported on 15 patients treated with CyberKnife radiosurgery for Obsessive-Compulsive Disorder (OCD) and depression with anxiety [11]. Limbic leucotomy was used for OCD, and subcaudate tractotomy with or without cingulotomy was performed for depression. Initial treatments used 75 Gy to the 80% isodose line with a 10 mm collimator, later reduced to 50 Gy to the 80% isodose line with a 7 mm collimator due to excessive necrosis. Among four patients with depression, median Hamilton Depression Scale scores dropped from 34 to 12, with three resuming their prior social lives. Although signal changes were observed on T2-weighted imaging at three months, specific targets and long-term follow-up data were not provided [11].

In 2019, Martínez-Álvarez reported treatment for patients with OCD, Depressive Disorder (DD), and Anorexia Nervosa (AN) with Gamma Knife RadioSurgery (GKRS) cingulotomy [4]. They delivered 120 Gy to the CB included in the 80% isodose region with two shots to each cingulum using a 4 mm collimator, with asymmetrical bilateral targets to avoid midline arterial exposure. Five patients with AN showed substantial body mass index improvements at six-month follow-up, and three patients with DD showed improvements in quality of life at two-year follow-up, with no reported adverse effects [4].

In 2024, Molina-Romero et al. used GKRS to target the anterior cingulate cortex bilaterally for intractable pain, using 120 Gy maximum dose, with two shots to each cingulum using a 4 mm collimator [9]. Three patients experienced 50-80% pain reduction without recurrence, while two had no improvement. Notably, four patients reported substantial reductions in anxiety and depression, and MRI at one year showed bilateral anterior CB hyperintensities consistent with ablative lesions [9].

This shows the therapeutic potential and safety of radiosurgical approaches to the CB and ACC in addressing either affective or pain-related disorders. Our case expands on this literature by demonstrating that a dual-targeted approach, addressing both medial thalamic and cingulum structures in a single session, can effectively treat chronic neuropathic pain and potentially improve comorbid anxiety-depressive disorder over time. Unlike prior studies that focused on single-pathology interventions, we highlight the feasibility and clinical benefit of combining these strategies, especially in highly complex, refractory cases such as the one presented. It is also important to highlight that this patient was treated with a single 4 mm shot with 90 Gy D Max to the CB bilaterally. At one year, both CBs showed the classical radiation lesion effect that would be expected at much higher doses. This cannot be underlined enough of the need to further study necrotic and sub-necrotic doses in functional radiosurgery.

Regarding target selection in thalamotomies, the centromedian-parafascicular (CM-Pf) complex plays a critical role in pain processing. The centromedian nucleus contributes to this function through its involvement in both pain perception and the affective dimension of pain, mediated by its extensive connections with the limbic system, through the medial pathway of pain. In contrast, the parafascicular nucleus may also contribute to pain modulation via its potential functional connection with the periaqueductal gray [14] that is implicated in the descending pathway of pain as well as the motor pathway (M1).

The ventral posteromedial nucleus (VPM) of the thalamus functions as a key relay center for afferent sensory input originating from the trigeminal principal sensory nucleus and spinal trigeminal nucleus. These signals are subsequently transmitted to the primary somatosensory cortex (S1) for processing. In addition to this primary pathway, the VPM also projects to contralateral intralaminar thalamic nuclei via the reticular formation. This widespread distribution of nociceptive signals across thalamocortical and subcortical regions contributes to the diffuse, poorly localized nature of chronic pain [14]. The VPM is part of the lateral pathway of pain.

Moreover, the medial and lateral pain systems are associated with distinct thalamic pathways. The medial system primarily involves the intralaminar thalamic nuclei, while the lateral system is linked to the ventral posterolateral (VPL) and ventral posteromedial (VPM) nuclei. This anatomical distinction underlies the differential processing of the affective-motivational and sensory-discriminative components of pain [27]. This is relevant in target selection, as involvement of all three pain pathways in the medial thalamus is possible with lower isodose lines, such as the 20 Gy, and may result in better clinical outcomes (In press).

Our group’s most recent publication by Del Cid et al. reported a retrospective analysis of 56 patients who underwent radiosurgery targeting the medial thalamus for refractory neuropathic or mixed cancer-related pain [14]. Depending on the clinical context, patients received either unilateral or bilateral thalamotomy. Del Cid et al. reported significant outcomes, with 66.6% of patients with oncologic pain and 71.7% of those with non-oncologic pain achieving at least 50% pain relief. In terms of radiomodulation effects, two out of two patients who underwent bilateral thalamotomy experienced a radiomodulatory response, compared to only one out of nine patients treated with unilateral thalamotomy [14]. Notably, bilateral thalamotomy demonstrated the fastest median time to pain relief (1.5 days), as compared to the triple-target treatment including the hypophysis (2.5 days), dual-target treatment (three days), and single thalamotomy (33 days) [14]. These findings underscore the potential benefits of a multi-target strategy tailored to specific pain characteristics and the possibilities of obtaining quick, substantial pain relief, as it was also experienced by this patient.

In our case, we treated dual pathology, chronic neuropathic pain, and anxiety-depressive disorder, through combined CB and medial thalamus targeting. The patient experienced sustained quality of life improvement, reduced emergency visits, and diminished opioid use, as well as functional independence, including return to work and daily activities. Although depressive symptoms persisted, requiring pharmacologic adjustment, the overall outcome was favorable.

Our treatment delivered asymmetric bilateral 90.0 Gy shots to the CB, minimizing exposure to anterior cerebral artery branches, and symmetric 120.0 Gy shots to the medial thalamus. All shots were administered using a 4.0 mm collimator during a single session. Notably, although the 90.0 Gy dose was intended as a sub-ablative and neuromodulatory dose, as described in other radiosurgical approaches [14], an MRI at 12 months showed contrast-enhancing lesions in the CB bilaterally, suggesting actual ablation and the classical disrupting effect of cingulotomy. Additionally, the left thalamic lesion appeared larger than expected, as shown in previous images (Figure 1); nevertheless, the patient did not present any new neurological deficit and was classified as an asymptomatic radiological adverse event.

Interestingly, our patient presented a unilateral hyper-responsiveness in the left hemisphere, despite bilateral irradiation with matched dosing and volumes. Interindividual variability in radiation sensitivity is well documented and may be influenced by genetic factors [28]. However, in our case, the asymmetry was not generalized but rather localized to the left hemisphere, suggesting a regional or locational factor. Similarly, animal studies have demonstrated heterogeneous regional brain responses to radiation due to differences in vascular density, cellular composition, and microglial activation [29]. In our case, this is a more possible causation, but still, something to consider is that we have evidence of different responses comparing the same brain regions to their contralateral pair.

Diffusion tensor imaging studies have shown a leftward asymmetry in fractional anisotropy within the CB, suggesting structural differences between hemispheres [30,31]. This asymmetry may partly explain the unilateral lesioning effect observed and supports the idea that the brain should be viewed not as a bilaterally symmetric organ, but as a structure with functional and structural lateralization. However, what has been mentioned above are some hypothetical considerations that may play a role in specific regional or locational responses to radiation, and should be taken with caution.

Nevertheless, these findings require further investigation into individualized radiosurgical dosing, predictors of hyper-responsiveness, and hemispheric susceptibility. While our case alone is insufficient to draw definitive conclusions, it shows the need for larger studies and extended follow-up to better understand radiation-induced effects in healthy brain tissue, as it is habitual in functional radiosurgery.

## Conclusions

Combined Gamma Knife cingulotomy and medial thalamotomy using quadruple-target radiosurgical planning could represent a clinical option for patients with severe, refractory neuropathic pain. Our original intention was to alter both limbic and thalamic neuronal circuitry to address chronic pain and modulate comorbid affective disorders, in particular anxiety. At 12 months post-treatment, we could not demonstrate improvement in anxiety-depressive symptoms; however, longer-term follow-up is needed to draw definitive conclusions.

The treatment dose, volume, and target location are topics that warrant additional investigation. In the meantime, what we know from our experience is that a 90.0 Gy dose can be ablative. Lower radiation doses took more time to produce a necrotic effect as opposed to 120 Gy at the thalamus in this patient, and it is aligned with current understanding of radiobiology. Special consideration regarding dosing strategies with lower doses in non-terminal patients experiencing refractory pain is warranted. This case adds new evidence supporting the safety and efficacy of Gamma Knife radiosurgery for complex pain management, while also prompting future research in connectomic-based SRS and dose-response variability.
